# Acupuncture Treatment Reverses Retinal Gene Expression Induced by Optic Nerve Injury via RNA Sequencing Analysis

**DOI:** 10.3389/fnint.2019.00059

**Published:** 2019-10-16

**Authors:** Jie Chen, Li Zhang, Lanying Liu, Xueqin Yang, Fengzhi Wu, Xiulun Gan, Rong Zhang, Yinjia He, Qiuyi Lv, Haonan Fu, Ling Zhou, Jiaxi Zhang, Anming Liu, Xiaodong Liu, Linqing Miao

**Affiliations:** ^1^School of Acupuncture-Moxibustion and Tuina, Beijing University of Chinese Medicine, Beijing, China; ^2^The Third Affiliated Hospital, Beijing University of Chinese Medicine, Beijing, China; ^3^School of Traditional Chinese Medicine, Beijing University of Chinese Medicine, Beijing, China; ^4^Journal Center, Beijing University of Chinese Medicine, Beijing, China; ^5^Beijing Advanced Innovation Center for Intelligent Robots and Systems, Beijing Institute of Technology, Beijing, China

**Keywords:** optic nerve crush, retinal ganglion cells, acupuncture, RNA sequencing, transcriptome, GB20 (Fengchi), BL1 (Jingming), GV16 (Fengfu)

## Abstract

Glaucoma and traumatic optic nerve crush (ONC) injury result in progressive loss of retinal ganglion cells (RGCs) and defects in visual function. In clinical trials of Traditional Chinese Medicine, acupuncture has been widely used for the treatment of ocular diseases. However, the molecular mechanisms of acupuncture treatment are still unclear. In this study, we used technique of RNA sequencing (RNA-seq) to study the effects of acupuncture treatment on retinal transcriptome after axotomy injury. RNA-seq results revealed that 436 genes including 31 transcription factors (TFs) were changed after injury, among them were many well-known neural degeneration related TFs such as Jun, Ddit3, Atf3, and Atf4. Interestingly, acupuncture treatment at acupoint GB20 (Fengchi) significantly reversed a series of differential expressed genes (DEGs) induced by optic nerve injury. While treatments at BL1 (Jingming) or GB20 sham control acupoint-GV16 (Fengfu), led to limited DEG reversal. In contrast, treatments at these two sites further enhanced the trend of DEG expression induced by axotomy injury. At last, retina immunostaining results revealed that only GB20 acupoint treatment increased RGC survival, in consistent with RNA-seq results. Therefore, our study first reported that acupuncture treatment regulated retinal transcriptome and reversed the gene expression induced by axotomy injury, and GB20 acupoint treatment increased RGC survival, which will provide novel therapeutic targets for treatment of ocular diseases.

## Introduction

Chronic eye diseases such as glaucoma and optic neuritis result in progressive loss of retinal ganglion cells (RGC) and blindness finally (Shindler et al., 2008; Almasieh et al., 2012). Traumatic injury such as optic nerve crush (ONC) also cause serious RGC death (Sanchez-Migallon et al., 2016). Previous studies show that both chronic and traumatic optic nerve injury induce endoplasmic reticulum (ER) stress in RGC soma, while manipulation of ER stress pathway promoted the survival of RGC (Yang et al., 2016; Huang et al., 2017). The induction of upregulation of pro-apoptotic TFs such as JUN and DDIT3 after injury leads to progressive RGC death (Huang et al., 2017; Syc-Mazurek et al., 2017a, b; Welsbie et al., 2017). Acupuncture has been widely used to treat many kinds of pains such as neck and shoulder pain, lower back pain worldwide (MacPherson et al., 2017; Koh et al., 2018). Acupuncture has also been used for treatment of ocular diseases, including glaucoma, age-related macular degeneration (AMD), retinitis pigmentosa, etc (Jiao, 2011; Xu et al., 2012; Law and Li, 2013; Xu and Peng, 2015). In Traditional Chinese Medicine, GB20 and BL1 are the common acupoints selected for acupuncture treatment of ocular diseases (Qin et al., 2015; Xu and Peng, 2015). Although acupuncture is widely used for ocular therapy, the effect of acupuncture treatment on regulation of retinal transcriptome especially those changed by ONC injury is totally unknown. RNA sequencing (RNA-seq) is a new technique effective in identifying numerous genes regulated by specific treatment. In this study, we will use ONC crush injury mouse model and RNA-seq technique to analyze retinal samples with and without optic nerve injury and screen for the upregulated and downregulated TFs 2 days after ONC injury, and also identified those injury-induced genes reversed after acupuncture treatment at two different acupoints.

## Results

### Expression Analysis

**FIGURE 1:**
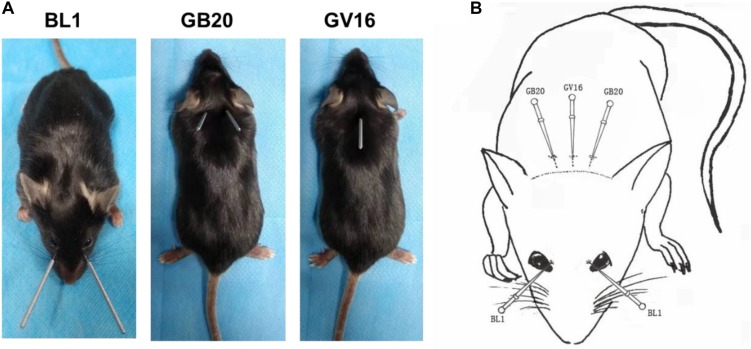
The acupoints for acupuncture treatments. **(A)** Photos of mice showing the sites of acupuncture treatments at acupoints BL1, GB20, and GV16, respectively. **(B)** Schematic image demonstrating acupoint sites on mouse.

To investigate expression differentiation, we extract RNA from intact retina (WT), axotomized retina (ONC), and axotomized retina with acupuncture treatment at acupoint GB20 (ONC-F) or BL1 (ONC-J), 2 days after ONC. The actual and schematic acupuncture sites were shown in Figures 1A,B. RNA integrity of each sample was assessed by Bioanalyzer 2100 system. Sample total reads ranged from 40.8 to 69.5 million, and mapping rate ranged from 95.4 to 96.6%.

**FIGURE 2:**
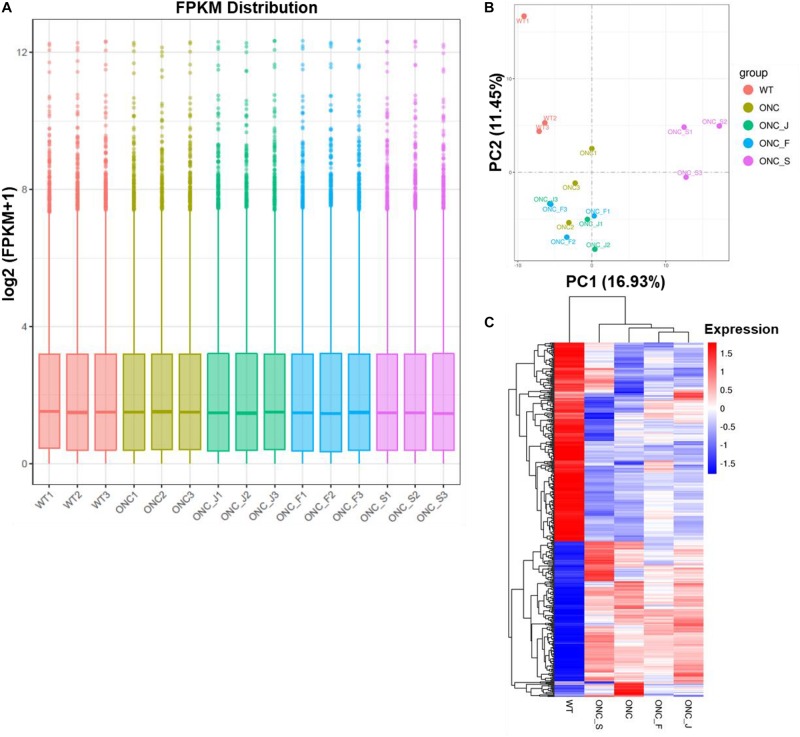
Quantitative analysis of retinal gene expression. **(A)** Boxplot shows the overall range and distribution of FPKM value of gene expression of all the samples. **(B)** PCA analysis shows the differentiation among samples. **(C)** Cluster analysis of differentially expressed genes among samples and groups. The color of the heat map indicated the relative gene expression. The deeper red color indicates the higher gene expression, while the deeper blue color indicates the lower gene expression.

We then used feature Counts v1.5.0-p3 to calculate expected number of Fragments Per Kilobase of transcript sequence per Millions base pairs sequenced (FPKM) of each sample, which represent relative gene expression abundance. The boxplot result showed that the overall distribution of the FPKM values were consistent among samples, suggesting that the RNA-seq data were reproducible (Figure 2A). Principal component analysis (PCA) showed that wild-type (WT) control formed clear cluster apart from injury groups, and ONC with or without acupuncture treatment formed one big cluster, ONC with sham treatment was separated from acupuncture treatment groups (Figure 2B). Differentially expressed genes (DEG) cluster analysis showed that acupuncture treatment groups were clustered together and separated from ONC control, and all the ONC groups with or without acupuncture treatments were separated from WT control group (Figure 2C).

### Differentially Expressed Genes After ONC

**FIGURE 3:**
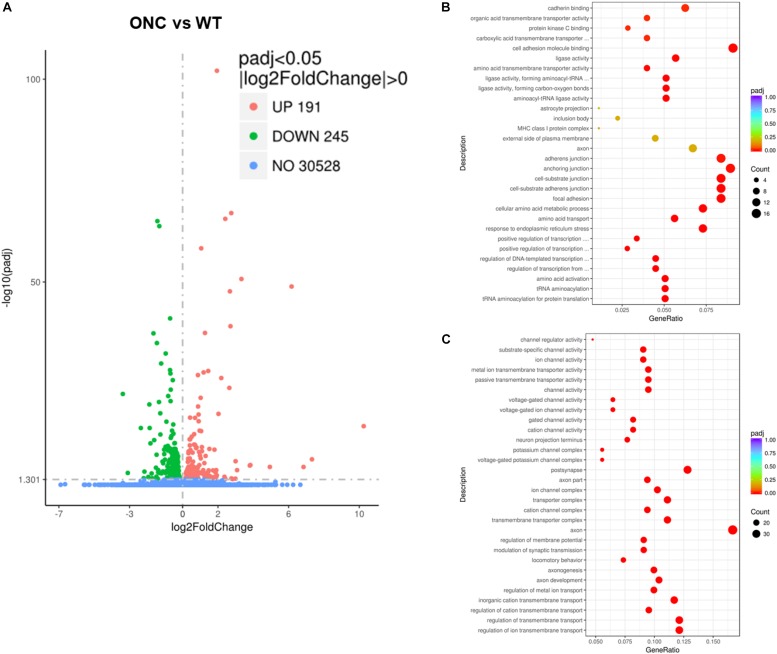
Differentially expressed genes induced by ONC injury. **(A)** The volcano map shows the overall differentially expressed genes after ONC injury, compared with WT control. **(B)** Enriched gene ontology analysis of up-regulated DEGs. **(C)** Enriched gene ontology analysis of down-regulated DEGs. padj: adjusted *p* value.

To identify candidate genes regulated by axon injury 2 days after axotomy, we performed differential expression analysis using the DESeq2 R package (1.16.1). Genes with | log_2_FoldChange| > 0 and adjusted *p*-value < 0.05 were assigned as differentially expressed. We then identified total 436 differentially expressed genes (DEG), with 191 genes upregulated and 245 genes downregulated (Figure 3A). Enriched gene ontology analysis results showed that the most upregulated gene categories were cell adhesions and junctions (Figure 3B); while the most downregulated gene categories were: axon, postsynapse and transmembrane transports (Figure 3C). The 20 most significantly upregulated and downregulated DEGs after ONC are shown in Table 1.

**TABLE 1:** Top 20 upregulated and 20 downregulated genes induced by ONC injury.

**Gene name**	**log_2_**	**Padj**	**Gene id**	**Gene description**
	**FoldChange**			
**Up-regulated**				
Sprr1a	10.2	3.6E-15	ENSMUSG00000050359	Small proline-rich protein 1A [Source:MGI Symbol;Acc:MGI:106660]
Gm29374	7.3	5.3E-07	ENSMUSG00000099839	Predicted gene 29374 [Source:MGI Symbol;Acc:MGI:5580080]
Gm38403	6.8	4.1E-05	ENSMUSG00000112854	Predicted gene, 38403 [Source:MGI Symbol;Acc:MGL:5621288]
Ecel1	6.7	3.8E-84	ENSMUSG00000026247	Endothelin converting enzyme-like 1 [Source:MGI Symbol;Acc:MGI:1343461]
Mmp12	6.2	1.3E-49	ENSMUSG00000049723	Matrix metallopeptidase 12 [Source:MGI Symbol;Acc:MGI:97005]
Cox6a2	4.9	4.0E-05	ENSMUSG00000030785	Cytochromec oxidase subunit 6A2 [Source:MGI Symbol;Acc:MGI:104649]
Cyb5r2	3.8	1.4E-05	ENSMUSG00000048065	Cytochromeb5 reductase 2 [Source:MGI Symbol;Acc:MGI:2444415]
Tmc1	3.8	1.8E-05	ENSMUSG00000024749	Transmembrane channel-like gene family 1 [Source:MGI Symbol;Acc:MGI:2151016]
Chac1	3.3	1.9E-51	ENSMUSG00000027313	ChaC, cation transport regulator 1 [Source:MGI Symbol;Acc:MGI:1916315]
Gm47593	3.1	2.5E-04	ENSMUSG00000112058	Predicted gene, 47593 [Source:MGI Symbol;Acc:MGI:6096640]
AC167169.1	3.0	1.6E-06	ENSMUSG00000117324	Novel transcript, antisense to Dscam
Plaur	3.0	3.2E-02	ENSMUSG00000046223	Plasmnogen activator urokinase receptor [Source:MGI Symbol;Acc:MGI:97612]
Top2a	2.8	4.0E-02	ENSMUSG00000020914	Topoisomerase (DNA) II alpha [Source:MGI Symbol;Acc:MGI:98790]
Hmox1	2.8	2.8E-02	ENSMUSG00000005413	Heme oxygenase 1 [Source:MGI Symbol;Acc:MGI:96163]
Atf3	2.8	1.0E-67	ENSMUSG00000026628	Activating transcription factor3 [Source:MGI Symbol;Acc:MGI:109384]
Hrk	2.7	8.2E-40	ENSMUSG00000046607	Harakiri, BCL2 interacting protein (contains only BH3 domain) [Source:MGI Symbol;Acc:MGI:1201608]
Sox11	2.7	2.0E-48	ENSMUSG00000063632	SRY (sex determining region Y)-box 11 [Source MGI Symbol;Acc:MGI:98359]
Cdkn1a	2.6	1.3E-24	ENSMUSG00000023067	Cyclin-dependent kinase inhibitor 1A (P21) [Source:MGI Symbol;Acc:MGI:104556]
Lad1	2.4	3.6E-02	ENSMUSG00000041782	Ladinin [Source:MGI Symbol;Acc:MGI:109343]
Tnfrsf12a	2.4	2.7E-66	ENSMUSG00000023905	Tumor necrosis factor receptor superfamily, member 12a [Source:MGI Symbol:Acc:MGI:1351484]
**Down-regulated**				
Scn4b	–3.38	4.1E-23	ENSMUSG00000046480	Sodium channel type IV beta [Source MGI Symbol;Acc:MGI:2687406]
Ppp1r1c	–3.10	1.2E-03	ENSMUSG00000034683	Protein phosphatase 1, regulatory inhibitor subunit 1C [Source:MGI Symbol;Acc:MGI:1923185]
Irx4	–2.36	1.0E-14	ENSMUSG00000021604	Iroguois homeobox 4 [Source:MGI Symbol;Acc:MGI:1355275]
Gpr101	–2.20	2.4E-02	ENSMUSG00000036357	G protein-coupled receptor 101 [Source MGI Symbol;Acc:MGI:2685211]
Hapln1	–2.16	5.7E-04	ENSMUSG00000021613	Hyaluronan and proteoglycan link protein 1 [Source:MGI Symbol;Acc:MGI:1337006]
Efcab1	–1.94	1.2E-02	ENSMUSG0C0O0068617	EF-hand calcium binding domain 1 [Source:MGI Symbol;Acc:MGI:1914043]
Pou4f2	–1.88	1.6E-20	ENSMUSG00000031688	POU domain, class 4, transcnption factor 2 [Source:MGI Symbol;Acc:MGI:102524]
Htr1b	–1.86	1.0E-14	ENSMUSG00000049511	5-hydroxytryptamine (serotonin) receptor IB [Source:MGI Symbol;Acc:MGI:96274]
Ctxn3	–1.82	6.2E-11	ENSMUSG00000069372	Cortexin 3 [Source:MGI Symbol;Acc:MGI:3642816]
Kctd19	–1.75	1.6E-03	ENSMUSG00000051648	Potassium channel tetramerization domain containing 19 [Source:MGI Symbol;Acc:MGI:3045294]
D130079A08Rik	–1.72	2.5E-02	ENSMUSG00000115424	RIKEN cDNA D130079A08 gene [Source:MGI Symbol;Acc:MGI:2444500]
Tusc5	–1.65	4.7E-38	ENSMUSG00000046275	Tumor suppressor candidate 5 [Source:MGI Symbol;Acc:MGI:3029307]
Pvalb	–1.61	9.8E-12	ENSMUSG00000005716	Parvalbumin [Source:MGI Symbol;Acc:MGI:97821]
2600014E21Rik	–1.52	4.2E-02	ENSMUSG00000100303	RIKEN cDNA 2600014E21 gene [Source:MGI Symbol;Acc:MGI:1919384]
Irx2	–1.51	6.3E-05	EMSMUSG00000001504	Iroguois homeobox2 [Source:MGI Symbol;Acc:MGI:1197526]
Tbx20	–1.48	1.0E-02	ENSMUSG00000031965	T-box 20 [Source:MGI Symbol;Acc:MGI:1888496]
Isl2	–1.47	4.0E-08	ENSMUSG00000032318	Insulin related protein 2 (islet 2) [Source:MGI Symbol;Acc:MGI:109156]
Tppp3	–1.45	1.1E-35	ENSMUSG00000014846	Tubulin polymerization-promoting protein family member3 [Source:MGI Symbol;Acc:MGI:1915221]
Sncg	–1.43	9.8E-66	ENSMUSG00000023064	Synuclein, gamma [Source:MGI Symbol;Acc:MGI:1298397]
Hydin	–1.40	3.4E-02	ENSMUSG00000059854	HYDIN, axonemal central pair apparatus protein [Source:MGI Symbol;Acc:MGI:2389007]

*Genes were selected by adjusted *p*-value < 0.05, |log_2_FoldChange| > 0 and FPKM > 0.*

### Transcription Factors Up-Regulated After ONC

Since TFs play important roles in both axon development and injury-induced degeneration, to narrow down the candidate DEGs, we next identified TFs upregulated and downregulated after axon injury, which result in 15 upregulated TFs and 16 downregulated TFs (Table 2). The upregulated TFs include many pro-apoptotic molecules involved in ER stress response, such as Atf4 and Ddit3 (Woo et al., 2012; Hiramatsu et al., 2014; Joo et al., 2015; Walter et al., 2018); Creb5 and Kdr in PI3K-Akt signaling pathway (Bellon et al., 2010; Kay et al., 2013; Wu et al., 2017); Jun and Cebpb in TNF signaling pathway (Mayer et al., 2013; Cao et al., 2018); Ddit3, Atf4 and Jun in apoptosis pathway (Holland et al., 2016; Ishizawa et al., 2016; Syc-Mazurek et al., 2017b), and most of these upregulated TFs resulted in RGC death.

**TABLE 2:** Transcription factors regulated by ONC injury.

**Gene name**	**log_2_**	***p*-value**	**Gene id**	**Gene description**
	**FoldChange**			
Atf3	2.75	1.02E-67	ENSMUSG00000026628	Activating transcription factor3 [Source:MGI Symbol;Acc:MGI:109384]
Sox11	2.68	2.05E-48	ENSMUSG00000063632	SRY (sex determining region Y]-box 11 [Source:MGI Symbol;Acc:MGI:98359]
Arid5a	1.45	8.81E-29	ENSMUSG00000037447	AT rich interactive domain 5A (MRFl-like) [Source:MGI Symbol;Acc:MGI:2443039]
Egr3	1.29	1.58E-02	ENSMUSG00000033730	Early growth response3 [Source:MGI Symbol;Acc:MGI:1306780]
Atf5	1.19	1.99E-28	ENSMUSG00000038539	Activating transcription factor 5 [Source:MGI Symbol;Acc:MGI:2141857]
Creb5	1.17	2.48E-04	ENSMUSG00000053007	cAMP responsive element binding protein 5 [Source:MGI Symbol;Acc:MGI:2443973]
Cebpb	1.13	1.79E-03	ENSMUSG00000056501	CCAAT/enhancer binding protien (C/EBP), beta [Source [731 Symbo;Acc:MGI:88373]
Ddit3	0.87	9.19E-28	ENSMUSG00000025408	DNA-damage inducible transcript 3 [Source:MGI Symbol;Acc:MGI:109247]
Fosl2	0.65	7.05E-05	ENSMUSG00000029135	Fos-like antigen 2 [Source:MGI Symbol;Acc:MGI:102858]
Jun	0.46	3.36E-13	ENSMUSG00000052684	Jun proto-oncogene [Source:MGI Symbol;Acc:MGI:96646]
Klf6	0.46	5.76E-03	ENSMUSG00000000078	Kruppel-like factor 6 [Source:MGI Symbol;Acc:MGI:1346318]
Bcl6	0.46	3.35E-02	ENSMUSG00000022508	B cell leukemia/lymphoma 6 [Source:MGI Symbol;Acc:MGI:107187]
Tead3	0.38	1.20E-02	ENSMUSG00000002249	TEA domain family member 3 [Source:MGI Symbol;Acc:MGI:109241]
Bhlhe40	0.31	1.19E-02	ENSMUSG00000030103	Basic helix-loop-helix family, member e40 [Source:MGI Symbol;Acc:MGI:1097714]
Atf4	0.19	4.73E-02	ENSMUSG00000042406	Activating transcription factor 4 [Source:MGI Symbol;Acc:MGI:88096]
Tsc22d1	–0.19	1.32E-02	ENSMUSG00000022010	TSC22 domain family, member l [Source:MGI Symbol;Acc:MGL:109127]
Six3	–0.24	1.18E-02	ENSMUSG00000038805	Sine oculis-related homeobox 3 [Source:MGI Symbol;Acc:MGI:102764]
Sebox	–0.25	4.97E-02	ENSMUSG00000001103	SEBOX homeobox [Source:MGI Symbol;Acc:MGI:108012]
Myt1I	–0.26	3.18E-02	ENSMUSG00000061911	Myelin transcription factor 1ike [Source:MGI:Symbol;Acc:MGI:1100511]
Ebf1	–0.38	2.29E-02	ENSMUSG00000057098	Early B cell factor 1 [Source:MGI Symbol;Acc:MGI:95275]
Scrt1	–0.41	1.50E-07	ENSMUSG00000048385	Scratch family zinc finger 1 [SourceMGI Symbol:Acc:MGL:2176606]
Irx6	–0.56	3.33E-03	ENSMUSG00000031738	Iroguois homeobox 6 [Source:MGI Symbol;Acc:MGI:1927642]
Eomes	–0.67	1.58E-02	ENSMUSG00000032446	Eomesodermin [Source:MGI Symbol;Acc:MGI:1201683]
Zicl	–0.70	2.12E-02	ENSMUSG00000032368	Zinc finger protein of the cerebellum 1 [Source:MGI Symbol;Acc:MGI:106683]
Pou6f2	–0.84	3.05E-04	ENSMUSG00000009734	POU domain, class 6, transcriptionfactor 2 [Source:MGI Symbol;Acc:MGI:2443631]
Pou4f1	–0.92	2.79E-16	ENSMUSG00000048349	POU domain, class4, transcription factor 1 [Source:MGI Symbol;Acc:MGI:102525]
IsI2	–1.47	4.00E-08	ENSMUSG00000032318	Insulin related protein 2 (islet 2)[Source:MGI Symbol;Acc:MGI:109156]
Tbx20	–1.48	1.02E-02	ENSMUSG00000031965	T-box 20 [Source:MGI Symbol;Acc:MGI:1888496]
Irx2	–1.51	6.26E-05	ENSMUSG00000001504	Iroquois homeobox 2[Source:MGI Symbol;Acc:MGI:1197526]
Pou4f2	–1.88	1.561-20	ENSMUSG00000031688	POU domain, class4, transcription factor 2 [Source:MGI Symbol;Acc:MGI:102524]
Irx4	–2.36	1.02E-14	ENSMUSG00000021604	Iroguois homeobox 4 [Source:MGI Symbol;Acc:MGI:1355275]

*Transcription factors were selected by adjusted *p*-value < 0.05, |log_2_FoldChange| > 0 and FPKM > 0.*

### Reversal of Injury Induced DEGs by Acupuncture Treatments

**FIGURE 4:**
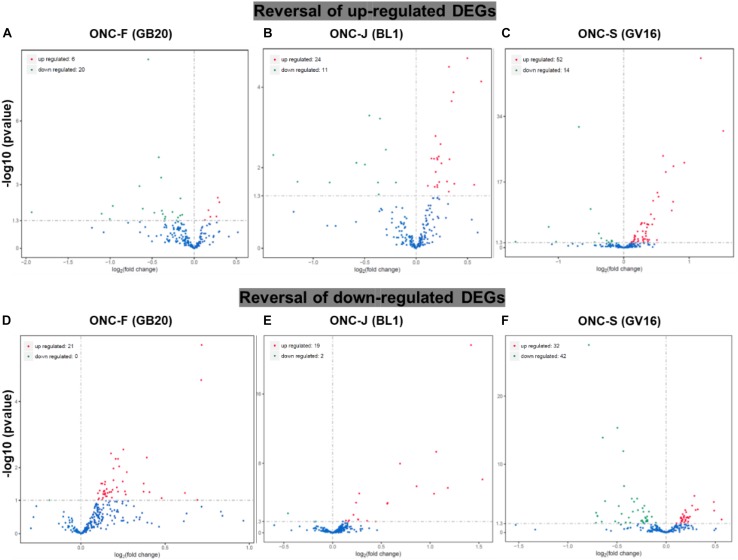
Reversal of DEGs by acupuncture treatment after ONC injury. **(A–C)** Volcano map shows the reversal of up-regulated DEGs induced by ONC injury after acupuncture treatments at acupoint BL1, GB20, and GV16 (Sham control site). **(D–F)** Volcano map shows the reversal of down-regulated DEGs induced by ONC injury after acupuncture treatments at acupoint BL1, GB20, and GV16. *p* value < 0.05.

To study the effect of acupuncture treatment after ONC injury, we performed acupuncture treatments 3 times after ONC at acupoint GB20 (Fengchi), BL1 (Jingming) or sham point GV16 (Fengfu) (Figure 1B), respectively. RNA-seq analysis showed that among the injury induced up-regulated DEGs, 20 DEGs were reversed by GB20 treatment with only 6 DEGs further up-regulated (Figure 4A), the reversal DEG number was 11 in BL1 and 14 in GV16 treatments, however, both BL1 and GV16 treatments further up-regulated even more DEGs, which was 24 in BL1 and 52 in GV16 (Figures 4B,C). Among the injury induced down-regulated DEGs, 21 DEGs were reversed by GB20 treatment without further down-regulated DEGs (Figure 4D), the reversal DEG number was 19 in BL1 with only 2 DEGs further down-regulated (Figure 4E), however, in GV16 treatments, the reversal number is 32 vs. 42 further down-regulated DEGs (Figure 4F). The average FPKM values of the 436 DEGs of all sample groups and the reversal percentages of all the DEGs after BL1, GB20, and GV16 treatments were listed in Supplementary Table S1.

### Validation DEGs With qRT-PCR

**FIGURE 5:**
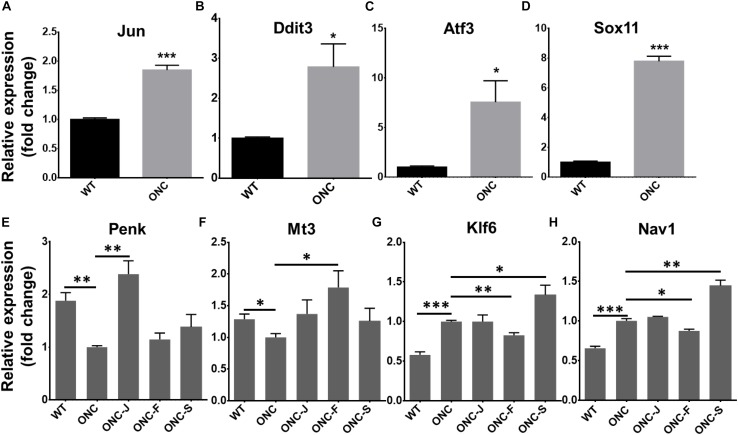
Validation of selected TFs and DEGs with qRT-PCR. **(A–D)** Statistical graph shows fold change of TFs mRNA expression, normalized to GAPDH level. **(E–H)** Statistical graph shows fold change of mRNA expression of DEGs reversed by acupuncture treatment, normalized to GAPDH level. Data are represented as means ± SEM; *n* = 3; ^∗^*p* < 0.05; ^∗∗^*p* < 0.01; ^∗∗∗^*p* < 0.001.

To verify the reliability of RNA-seq result, we selected four important TFs reported to be upregulated after axon injury (Jun, Ddit3, Atf3, and Sox11) (Hu et al., 2012; Fagoe et al., 2015; Holland et al., 2016; Huang et al., 2017; Lu et al., 2017; Norsworthy et al., 2017; Struebing et al., 2017; Li et al., 2018; Wong et al., 2018) to perform qRT-PCR test. Results showed that all the four TFs were significantly upregulated after ONC injury (Figures 5A–D), consistent with previous reports. We then also test the DEGs expression reversed by acupuncture treatment. Results showed that expression of Penk (Preproenkephalin) and Mt3 (Metallothionein-3) were significantly decreased after ONC injury, Penk was reversed by BL1 (ONC-J) treatment and Mt3 was reversed by GB20 (ONC-F) treatment, while sham treatment GV16 (ONC-S) had no effect on expression reversal of both candidate DEGs (Figures 5E,F). Expression of Klf6 (Kruppel-like factor 6) and Nav1 (neuron navigator 1) were significantly increased after ONC injury and only GB20 treatment reversed and decreased the expression of candidate genes, while BL1 treatment had no effect on expression change and GV16 sham treatment further increased the expression of candidate genes (Figures 5G,H). All these qRT-PCR results were consistent with RNA-seq analysis and demonstrated that acupuncture treatment regulated retinal transcriptome after ONC injury.

### Acupuncture Treatment Promote RGC Survival

**FIGURE 6:**
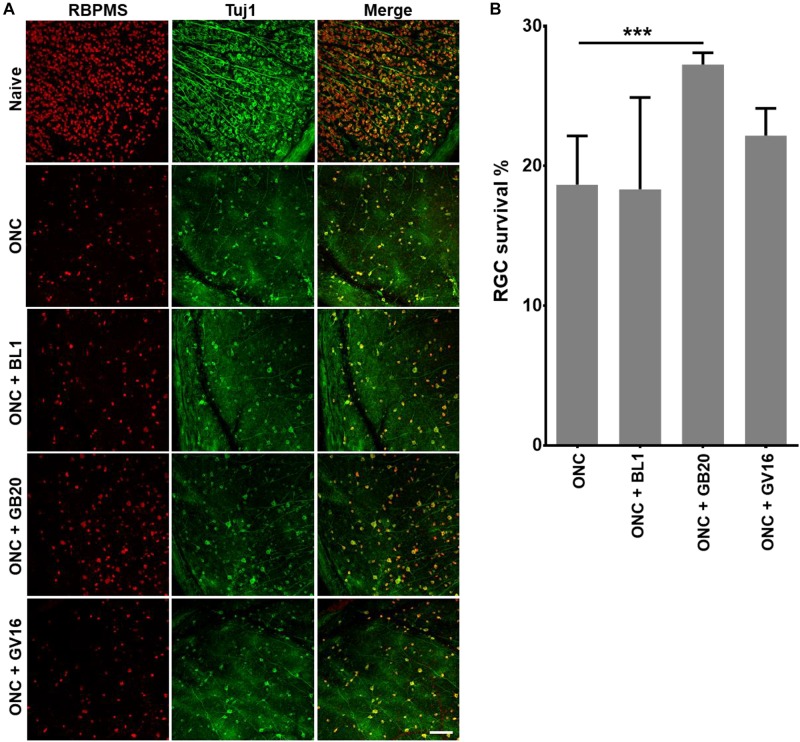
Promotion of RGC survival by acupuncture treatment. **(A)** Confocal images showing the co-staining of RGC with antibodies of RBMPS (RGC specific marker) and Tuj1 (neuron specific marker). **(B)** Statistical analysis of survival RGC percentage relative to intact naïve retina. Data are represented as means ± SEM; *n* = 5; ^∗∗∗^*p* < 0.001.

Finally, to test how acupuncture treatment affected actual survival of RCGs after ONC, we collected and immunostained the retinas after ONC alone and ONC with acupuncture treatment at acupoints BL1, GB20, and sham control acupoint GV16. Retinas were co-stained with RGC specific marker-RBPMS and neuron specific marker-Tuj1. Results showed that GB20 acupoint treatment slightly increased RGC survival after optic nerve injury. 27% of RCGs survived in GB20 acupuncture condition, while only 18% survived under ONC alone. BL1 and GV16 had no significant effect on RGC survival, which was 18 and 22%, respectively (Figure 6). Thus, ONC with acupoint GB20 treatment resulted in 9% more RCGs surviving compared to ONC alone.

## Discussion

In this study, we performed RNA-seq analysis of retinal transcriptome 2 days after ONC.

Axotomy injury induced both upregulation and downregulation of more than eight hundred genes, including many pro-apoptotic molecules and ER stress pathway molecules. Our previous studies showed that both traumatic and chronic neuron injury resulted in activation of PERK-eIF2α-ATF4-CHOP pathway or downregulating activity of PI3K-AKT-mTOR pathways, while manipulated these pathways and reversed the injury induced changes resulted in neuroprotection and increased RGC survival (Park et al., 2008; Miao et al., 2016; Yang et al., 2016; Huang et al., 2017, 2019). DLK-JNK-JUN pathway is another important pathway activated by axon injury and knockdown this pathway also resulted in neuroprotection (Kim et al., 2016; Syc-Mazurek et al., 2017b).

In Traditional Chinese Medicine, acupuncture has been broadly used for treatment of ocular diseases. GB20 and BL1 are the most common acupoints for ocular disease treatment. However, BL1 acupoint is too close to the eyeball and it’s difficult to handle during clinical practice, and it is the same in mouse acupuncture treatment (Figure 1); GB20 acupoint has many muscles surrounded and it is easy to perform needling at this acupoint. RNA-seq analysis revealed that acupuncture treatment at GB20 reversed the expression of 41 genes and only 6 genes were regulated toward the same direction induced by ONC injury (Figures 4A,D). In BL1 acupoint treatment, 43 genes were reversed, and 13 genes were regulated toward the same direction induced by ONC injury (Figures 4B,E). Although sham treatment at GV16 also reversed 46 genes, 94 genes were regulated toward the same direction induced by ONC injury (Figures 4C,F). As in qRT-PCR results, sham treatment further increased Klf6 and Nav1 expression which were already increased by ONC injury (Figures 5G,H). Therefore, only GB20 treatment had the best overall reversal effect on DEGs expression, and GB20’s sham control GV16 treatment had no overall reversal effect at all, in contrast, sham treatment further enhanced the expression states induced by ONC injury. What is more, the promotion of RGC survival by GB20 treatment confirmed the neuroprotection effect of DEG reversal (Figure 6B).

Among these reversal DEG candidates, Mt3 plays an important role in ocular neovascularization and its deficiency will exacerbate retinal degeneration (Tsuruma et al., 2012; Choi et al., 2013); Fbn1 (fibrillin 1) expression is required for eye development and its mutation is associated with macular degeneration (Hubmacher et al., 2014; Ratnapriya et al., 2014), suggesting that acupuncture treatment may affect vascularization processes in the retina, and promote neuroprotective outcomes after ONC injury. Intriguingly, the site of acupoint GB20 is at the back of the neck, far from the site of eyes, while needling and stimulation at this point surprisingly has effect on regulation of retina gene expression, which explains why many clinical practices choose GB20 site to cure ocular diseases. At last, it is possible that acupuncture treatments relay electrical signal via dorsal root ganglion (DRG) and spinal cord, finally regulate ocular blood flow since many groups reported that acupuncture treatment improved eye blood flow in open-angle glaucoma patients (Leszczynska et al., 2018; Vanzini and Gallamini, 2018). It is reported that acupuncture treatment also regulated expression of nerve growth factor and brain-derived neurotrophic factor in retina (Pagani et al., 2006) which may regulate retinal gene expression and provide neuroprotection. Since GB20 treatment slightly increase RGC survival, next we will investigate the detail mechanism under GB20’s involvement in RGC protection.

## Materials and Methods

### Animals

We perform experiments in 6 weeks old C57BL/6 mice. All animal procedures were performed in accordance with the National Institute of Health guidelines. The protocol was approved by the Animal Care and Use Committee of Peking University.

### Optic Nerve Crush

Mice were anesthetized by xylazine and ketamine based on their body weight (0.01 mg xylazine/g + 0.08 mg ketamine/g). Optic nerves of both sides were sequentially exposed intraorbitally and crushed with a jeweler’s forceps (Dumont #5; Fine Science Tools) for 2 s approximately 0.5 mm behind the eyeball. Care was taken not to damage the underlying ophthalmic artery. Erythromycin Eye Ointment was applied to protect the cornea after surgery.

### Acupuncture Treatment

After optic nerves crush injury, mice were anesthetized by xylazine and ketamine based on their body weight (0.01 mg xylazine/g + 0.08 mg ketamine/g) before acupuncture treatment at acupoint GB20 or BL1 at both sides, respectively. The depth of the acupuncture needling is around 2mm. The duration of acupuncture treatment was 20 min. We then gave another two acupuncture treatments every 24 h.

### RNA Preparation

Mice were randomly divided into four groups (3 mice/group). Experiments were repeated for 3 times. Briefly, in each replicate, mice were sacrificed 2 days after ONC injury, and retinas were dissected out in HBSS buffer (Cellgro) immediately. Retinas were then homogenized with TRIzol Reagent (Thermo Fisher Scientific), and total RNA was extracted from the homogenized mixture according to the reagent instructions. RNA purity was checked using the NanoPhotometer^®^ spectrophotometer (IMPLEN). RNA concentration was measured using Qubit^®^ RNA Assay Kit in Qubit^®^ 2.0 Flurometer (Life Technologies). RNA integrity was assessed using the RNA Nano 6000 Assay Kit of the Bioanalyzer 2100 system (Agilent Technologies).

### Library Preparation and Sequencing

About 3000 ng total RNA generated from each group was used for RNA-seq, which was done at Novogene, Inc. Briefly, RNA samples from three biological replicates went through mRNA purification with poly-T oligo-attached magnetic beads. Sequencing libraries were generated using NEBNext^®^ UltraTM RNA Library Prep Kit for Illumina^®^ (NEB) following manufacturer’s recommendations and index codes were added to attribute sequences to each sample. The library fragments were purified with AMPure XP system (Beckman Coulter) for cDNA fragments of preferentially 250∼300 bp in length. Library quality was assessed on the Agilent Bioanalyzer 2100 system. The clustering of the index-coded samples was performed on a cBot Cluster Generation System using TruSeq PE Cluster Kit v3-cBot-HS (Illumia) according to the manufacturer’s instructions. After cluster generation, the library preparations were sequenced on an Illumina Hiseq platform and 125 bp/150 bp paired-end reads were generated.

### Gene Expression Analysis

The RNA-seq reads were aligned to the reference genome using Hisat2 v2.0.5. FeatureCounts v1.5.0-p3 was used to count the reads numbers mapped to each gene. And then FPKM of each gene was calculated based on the length of the gene and reads count mapped to this gene, which normalizes gene expression by considering the effect of sequencing depth and gene transcript length at the same time. Differential expression analysis was performed using the DESeq2 R package (1.16.1). The resulting *p*-value were adjusted using the Benjamini and Hochberg’s approach for controlling the false discovery rate (less than 0.05). Genes with an adjusted *p*-value < 0.05 (detected by DESeq2) were considered to be differentially expressed.

### Quantitative Real-Time PCR (qRT-PCR)

Twelve RNA samples (3 samples each group) were examined with qRT-PCR. 1 μg total RNA from each sample was reverse-transcribed into cDNA using SuperScript III (Invitrogen). All qRT-PCR assays were performed in duplicate. Reactions were carried out with SYBR^®^ Premix Ex Taq^TM^ kit (TaKaRa) and performed on LightCycler^®^ 480 System (Roche). For comparison of relative gene expression, we analyzed qRT-PCR data by ΔCt method and normalized value to endogenous GAPDH control.

### Immunostaining of Flat-Mount Retina

Retinas were dissected out from 4% PFA fixed eyes and washed extensively in PBS before blocking in staining buffer (10% normal goat serum and 2% Triton X-100 in PBS) for 30 min. Mouse neuronal class ß-III tubulin (clone Tuj1, 1:200; Biolegend), rabbit RBPMS (GTX118619, 1:400, GeneTex) were diluted in the same staining buffer. Floating retinas were incubated with primary antibodies overnight at 4°C and washed three times for 30 min each with PBS. Secondary antibodies (Cy2 and Cy3-conjugated) were then applied (1:200; Jackson ImmunoResearch) and incubated for 1 h at room temperature. Retinas were again washed three times for 30 min each with PBS before a cover slip was attached with Fluoromount-G (Southernbiotech).

### Counting Surviving RGCs

For RGC counting, whole-mount retinas were immunostained with RBPMS antibody, and 4–6 fields were randomly sampled from peripheral regions of each retina. The percentage of RGC survival was calculated as the ratio of surviving RGC numbers in injured eyes compared to contralateral uninjured eyes.

### Statistical Analysis

Data are represented as means ± SEM. qRT-PCR data was analyzed with Student’s *t*-test, and *P*-value < 0.05 was considered as statistically significant. The raw data and GEO accession number for this study are as follows: GSE131486, link: https://www.ncbi.nlm.nih.gov/geo/query/acc.cgi?acc=GSE131486.

## Data Availability Statement

The datasets generated for this study can be accessed on NCBI website via GEO accession number: GSE131486.

## Ethics Statement

All animal procedures were performed in accordance with the National Institutes of Health guidelines.

## Author Contributions

LM and XL designed the experiments. LM, XL, JC, LZha, LL, XY, FW, XG, RZ, YH, QL, HF, LZho, JZ, and AL performed the experiments, and collected and analyzed the data. LM prepared the figures. LM and XL prepared the manuscript.

## Conflict of Interest

The authors declare that the research was conducted in the absence of any commercial or financial relationships that could be construed as a potential conflict of interest.
